# Supplementary material for: Spatiotemporal distribution characteristics of Nanjing place names—Based on data mining of Tang-Song poetry and online travelogues
Source: PLoS One. 2025 Feb 24;20(2):e0319244. doi: 10.1371/journal.pone.0319244 (PMC12005594; doi:10.1371/journal.pone.0319244)
Supplement: Attachment — Submitted filename: Response to Reviewers.docx [file pone.0319244.s004.docx]

**Response to Reviewers/Editors –** **PONE-D-24-55005**

Dear Reviewers/Editors,

We sincerely appreciate your coordination in arranging the review process for our manuscript titled "Spatiotemporal distribution characteristics of Nanjing place names" (Manuscript ID: PONE-D-24-55005) and thank the reviewers for their constructive feedback.

In response to the comments, we have carefully revised the manuscript and provided a detailed point-by-point response to each critique. All modifications are explicitly highlighted in the revised version annotated in yellow text for clarity. The specific revisions are outlined as follows:

Response to Journal Requirements

*1. In accordance with the journal's formatting guidelines, we have implemented comprehensive adjustments to the manuscript, including standardization of author affiliations, refinement of title phrasing, restructuring of section headings, normalization of reference list formatting style, and reorganization of supplementary appendices. These modifications ensure full compliance with the* *journal's technical specifications.*

*2. All research data generated or analyzed in this study, encompassing geographical nomenclature, geospatial coordinates, artwork metadata, and associated descriptors, will be archived as supplementary materials accompanying the final manuscript submission.*

*3.* *The financial disclosure section has been revised to fully comply with the journal's requirements. Specifically:*

*“This work was supported by the Ministry of Education Humanities and Social Sciences Foundation Project (Grant No. 24YJA790090). The funders had no role in study design, data collection and analysis, decision to publish, or preparation of the manuscript.”*

*4. The funding-related text has been removed from the manuscript.*

*5. All Supporting Information (SI) files containing the complete dataset necessary to replicate this study will be archived as supplementary materials and submitted concurrently with the main manuscript through the submission system.*

*6. The ORCID ID of corresponding author has been validated in Editorial Manager.*

*7. We confirm that Table 2 has been appropriately cited in the revised manuscript (Page 13, Line 403).* *We sincerely apologize for the previous oversight, which occurred due to an inadvertent formatting error during document conversion.*

*8. In response to the reviewers' comments, we have revised the reference list by selectively replacing outdated sources and incorporating recent seminal publications. All modifications are explicitly highlighted in the manuscript annotated in yellow text, and the citation numbering/style has been checked to ensure the compliance with the journal's formatting guidelines.*

*9. All figures have been submitted through the Preflight Analysis and Conversion Engine (PACE) system and validated for compliance with PLOS formatting standards, including resolution (600 dpi for line art), color mode (CMYK), and file type (TIFF).*

Response to Main Comments

Motivation

1) The authors provide a thorough explanation of the research gap. They highlighted that previous studies of spatiotemporal distributions have rarely approached the topic from a geographic perspective using extensive longitudinal data. However, the manuscript's comparative analysis between historical poetry and modern travel narratives would benefit from a clearer grounding. While the authors implicitly draw parallels between Tang-Song poets as travelers and contemporary tourists, this connection deserves more explicit development in the theoretical framework. The poets' dual role as both literary figures and travelers appears to be a key premise of the study, but this perspective needs to be more clearly articulated. The conclusion presents an intriguing argument about how comparing historical and contemporary urban travelers can offer novel insights into cities' cultural legacies. I wish the authors would introduce this valuable perspective earlier in the manuscript, ideally in the introduction, to better motivate the study.

- *We sincerely appreciate the reviewer's insightful observation regarding the dual roles of classical Chinese literati as both literary figures and travelers, which has profoundly informed our analytical framework. In the revised manuscript, we have explicitly articulated and substantiated this perspective through targeted textual expansions and conceptual clarifications.* *The comprehensive revisions addressing this perspective can be found in* ***Lines 39-42*** *and* ***Lines 75-84*** *of the revised manuscript.*

Literature review

1) Please briefly review prior studies examining contemporary urban travelers. This can allow the authors to highlight the lack of studies comparing historical and modern travelers.

- *In response to your recommendation, we have added Section “2.3 Urban Tourists” (****Lines 242-259)*** *to the literature review. This enhancement has substantially strengthened the theoretical foundation of our study.*

2) While the Chinese literary geography coverage is strong, consider briefly discussing similar work from other regions (UK, US, etc.) to provide international context.

- *We sincerely appreciate your suggestion. Our initial analysis indeed lacked a cross-cultural comparative perspective. To address this limitation, we have incorporated research findings on the relationship between British literary genres and their geographical determinants, thereby enhancing the international relevance of this study's scholarly contribution. The comprehensive revisions can be found in* ***Lines 117-125*** *and* ***Lines 139-143*** *of the revised manuscript.*

Methods

1) The study analyzed classical poetry as well as contemporary travel narratives. However, in the data sources and processing section, the study discusses in detail the collection of classical poetry and only mentions how modern travel narratives were collected on page 15. Please consider moving this discussion to the data preprocessing/methods section.

- *We have moved this discussion to the “3.1 Data Sources and Processing” section.* ***Lines 327-334.***

2) Briefly explain the process of excluding works that do not reference specific locations in Tang-Song poetry. Was this automated or manual?

- *In the Data Processing section, we have expanded the description of our methodological protocols for cross-referencing and exclusion criteria regarding artwork information.* ***Lines 271-273.***

3) P.8: The manuscript mentions that some places have undergone a spatial shift overtime. Please provide evidence for historical place name changes and spatial shifts.

- *In this section, we have incorporated additional case studies, key findings, and references drawing on the authoritative scholarship of prominent Chinese historians, which we believe will substantiate the arguments presented.* ***Lines 284-316.***

4) P.9: regarding this part “Emotion plays a critical role in classical Chinese poetry, serving as the subjective expression of the authors' perceptions of place. This emotional dimension provides valuable insights for studies in literary geography.” I believe discussing the significance of examining emotions here may be repetitive as the manuscript previously discussed this point in the literature review and introduction.

- We acknowledge the validity of your suggestion and have removed the redundant content in this section. ***Lines*** *320****-323.***

5) Why were three categories used to code emotions in this study? This decision was only well-motivated in the “research outcomes” section. It would be better to move this motivation earlier to the methods/data preprocessing section.

- *In Section 3.2 (Research Methods), we have added subsection 3.2.3 (Emotional Type Analysis) to provide a conceptual foundation for emotional categorization and related methodological considerations prior to discussing the results.* ***Lines 362-381.***

6) In the frequency analysis, consider using proportions instead of raw counts for better comparability.

- *In the frequency analysis, as shown in Table2 we have conducted proportions instead of raw counts for better comparability.* ***Lines 414.***

7) Address the sample size disparity between dynasties (109 Tang poems vs 199 Song poems).

- *We have provided contextualized descriptions and analytical evaluations of the historical context underlying the discrepancies in sample sizes. This enhancement aims to systematically elucidate the historical determinants contributing to such variations.* ***Lines 400-402; Lines 406-410.***

8) Please specify the emotion classification tool and its accuracy.

- *In Subsection 3.2.3 (Emotional Type Analysis) we have added an explanation to specify the emotion classification tool and its accuracy.* ***Lines 377-381.***

9) Consider how poetic techniques (imagery, allusions, metaphors) may affect emotion detection, particularly given the stylistic differences between Tang and Song poetry. Some studies support the distinct linguistic expression in Tang versus Song poetry (Yang, Zheng, & Shao, 2018).

- *In Section 3.2.3 (Emotional Type Analysis), we have expanded the discussion with necessary clarifications, addressed common classification methodologies and their application boundaries, and supplemented relevant citations to directly address your recommendation.* ***Lines 368-377.***

10) Account for how a poem's genre might have influenced emotional expression, and whether the genre effect may have played a role in the current findings.

- *In Section 3.2.3 (Emotional Type Analysis), we have expanded the discussion with necessary clarifications and supplemented relevant citations to directly address your recommendation.* ***Lines 363-368.***

Response to Minor Comments

1) Some terms are introduced without definitions. Incorporating the intended definition for each term will enhance the readability of the paper. The terms include: cultural landscapes, digital humanities techniques, and toponyms.

- *In accordance with the reviewers' suggestions, we have supplemented the definitions of key terminology in the text to facilitate readers' intuitive understanding of our conceptual framework. The specific enhancements include:* ***Lines 44-51****;* ***Lines 65-66****;* ***Line 110-112****.*

2) P.3: please cite studies which lie in this group to guide interested readers to the relevant literature: “The third area of focus examines the relationship between literary genres and geography”.

- *In response to the reviewers' recommendations, we have expanded the reference list to enhance readers' comprehension and facilitate further exploration of the perspectives presented.* ***Lines 115-117.***

3) P.6: The reader may not understand the relevance of “Marxist principles” in this sentence “classical poetry stands as a cornerstone of China’s rich traditional culture, and its study holds significant contemporary value for both cultural preservation and the integration of Marxist principles with traditional Chinese heritage”. Please clarify this phrase to enhance the coherence of the paper.

- *We appreciate your suggestion as both constructive and essential to strengthening the scholarly rigor of this work. To mitigate potential misinterpretation, we have revised the relevant phrasing as follows: In summary, classical poetry stands as a cornerstone of China’s rich traditional culture, and its study holds significant contemporary value for both cultural inheritance and cultural heritage utilization.* ***Lines 227-229.***

4) P.8: in the sentence “Given that the Tang and Song dynasties predate the Ming and Qing dynasties, “, the comparison with the Ming and Qing may not be necessary. The sentence may focus on how long ago the Tang and Song dynasties were, which could highlight the centuries of change these cultural locations have experienced.

- *We have revised the phrasing of this sentence to read as follows:* *Given that the Tang and Song dynasties are from a quite ancient period, the places mentioned in Tang and Song poetry have experienced more substantial changes over time. In some cases, these place names have been misinterpreted by later generations, leading to spatial shifts, with different locations sharing the same name.* ***Lines 281-284.***

5) Consider mentioning in the limitations that only poems that explicitly feature names were included in the analysis, which constituted a very small sample of Tang-Song poetry, while those which only described a cultural place were not included. This limitation may guide future research to explore a technique that addresses this issue.

- *In accordance with your recommendation, we have addressed the sample size limitations in the Discussion section (Section 5) and delineated this issue as a priority research direction for future investigations. Furthermore, we have incorporated your constructive suggestion into our team's research agenda framework. We sincerely appreciate your expertise in identifying this critical methodological dimension, which will continue to guide our ongoing scholarly explorations.* ***Lines 583-585.***

***We sincerely appreciate the valuable feedback and constructive suggestions provided by the reviewers and editors, and we deeply value their expertise and thoughtful consideration throughout the revision process.***
